# Focus on sex hormone axis disorder: exploring the susceptibility mechanism of dry eye in perimenopausal women

**DOI:** 10.3389/fmed.2026.1788556

**Published:** 2026-03-16

**Authors:** Manhui Zhu, Yufei Wang, Yuhang Na, Yuanyuan Tu, E. Song

**Affiliations:** 1 Department of Ophthalmology, Lixiang Eye Hospital of Soochow University, Suzhou, Jiangsu, China; 2 Department of Pathogen Biology, Medical College, Nantong University, Nantong, Jiangsu, China

**Keywords:** dry eye, hormone replacement therapy, pathogenesis, perimenopause, risk factors, sex hormone

## Abstract

Dry eye (DE) is a disease that affects the ocular surface in multiple aspects. The main feature of this disease is the disruption of tear film balance. If not effectively controlled, DE can cause ocular discomfort, pain and visual impairment. These problems will seriously affect patients’ quality of life. Perimenopausal women are at extremely high risk of DE, and their pathogenesis and risk factors have distinct stage specificity. The core view is that the disorder of sex hormones caused by ovarian failure is the initial and core link leading to the imbalance of tear film homeostasis. On this basis, chronic immune inflammatory response, neuroendocrine changes, perimenopausal depression, sleep disorders and the use of specific drugs such as hormone replacement therapy (HRT), as important contributing factors, further amplify the risk of disease in this group. Through a comprehensive analysis of these multi-level and intertwined risk factors, this review emphasizes the need for individualized management strategies for the prevention and treatment of DE in perimenopausal women, so as to provide theoretical basis and new ideas for the standardized clinical diagnosis and treatment of DE in perimenopausal women.

## 1 Introduction

Dry eye (DE) is a complex disease involving multiple aspects. It has now become a very common and highly prevalent disease, posing a significant threat to global eye health (1). The incidence of DE is not fixed; it varies within the range of 5 to 50% and shows an upward trend as people age (2, 3). DE can present with a variety of clinical manifestations. These symptoms include a feeling of dryness in the eyes, itching, a sensation of foreign objects in the eyes, a burning sensation, disliking light, and blurred vision (2). In some particularly extreme cases, there may be a decline in visual clarity. Long-term eye discomfort and its negative impact on work and life may lead to anxiety, depression and sleep disorders, which seriously affect the physical and mental health of patients (4). As early as 2011, the annual medical expenditure on DE in the United States reached 3.8 billion US dollars. A large number of direct and indirect costs related to DE make it a serious public health problem and socioeconomic burden (5).

The tendency that Asian ethnic groups are prone to DE has been widely recognized, the prevalence of DE in East Asian populations (16.7–33.4%) is significantly higher than that in Caucasians (8.7–11.0%) (6, 7). Differences in prevalence of DE across multiple population cohorts were no longer significant after adjustment for lifestyle factors, suggesting that factors such as diet, indoor humidity, and screen exposure play an important role in the pathogenesis of DE (7). Additionally, age and gender have consistently been identified as independent risk factors for DE in multiple studies. Women are generally considered to be the high-risk group for DE. In all age groups, the proportion of women suffering from DE is higher compared to men of the same age, especially in women over 50 years old (8). Among people over 50 years old in the United States, the prevalence rate of DE among women is approximately twice that of men of the same age group (15.2% in women, 7.0% in men) (9). A similar trend has been observed in Asian populations, and the severity of DE increases with age (10). Since most women enter perimenopausal period around the age of 50, this difference in prevalence of DE may be regulatory effects of hormones and hypothalamic–pituitary hormones on the Lacrimal Functional Unit (LFU) in perimenopausal women, as well as their regulatory effects on the immune system (11, 12).

However, studies on the pathogenesis and treatment of DE in perimenopausal women remain limited (13). This review systematically reviews relevant literature at home and abroad. It aims to summarize the complex relationship between physiological changes in perimenopausal women and the pathological mechanism of DE. It is expected to provide useful references for further exploring the pathogenesis of DE in perimenopausal women and developing more effective treatment strategies.

## 2 Pathophysiological basis of dry eye and perimenopause

DE is an ocular surface disease caused by multiple factors. The main feature of this disorder is the loss of tear film homeostasis, accompanied by various ocular surface symptoms (1). The pathogenesis of DE includes tear film instability, tear hyperosmolarity, ocular surface damage, inflammation, and abnormal neural perception (1). To maintain the health of the ocular surface, it largely depends on a healthy tear film structure. The tear film is a liquid layer with a thickness of approximately 3 micrometers, composed of different elements such as proteins, lipids, metabolic residues, and electrolytes. The tear film has several crucial functions such as lubricating the ocular surface, transporting oxygen and nutrients, and providing a refractive medium (14). Structurally, the tear film is composed of three layers, which are arranged in sequence from the outermost layer to the innermost layer. The outermost layer is the lipid layer, which is produced by the meibomian glands. The middle layer is the aqueous layer, which is formed by the secretions of the lacrimal glands and accessory lacrimal glands. The innermost layer is the mucin layer, which is secreted by conjunctival goblet cells and some other cell type (15). The loss of tear film homeostasis, a central driver in the pathophysiology of DE, predominantly results from dysfunction in one or more ocular structures responsible for producing and/or regulating tear film components (16).

From the perspective of etiology, DE can be classified into three relatively main types, namely water deficiency dry eye, evaporative dry eye and mixed dry eye. It should be noted that these classifications are not completely separate or unrelated. In fact, they often overlap (17). The main cause of water deficiency dry eye is insufficient tear secretion. This insufficient secretion is the result of lacrimal gland dysfunction (18). Evaporative dry eye is the most common type of DE, which is mainly related to abnormal lipid layer and excessive tear evaporation caused by meibomian gland dysfunction (MGD) (19). Moreover, recent evidence suggests that aqueous deficiency may also contribute to evaporative dry eye, as reduced tear volume can exacerbate tear film instability and hyperosmolarity (20). Both reduced secretion and excessive evaporation can lead to increased tear osmolarity. High-osmolarity tears directly damage the ocular surface. At the same time, they release pro-inflammatory factors. This release induces apoptosis of corneal epithelial cells and leads to a decrease in goblet cells. Thus, a vicious circle of “hyperosmolarity-inflammation-damage” is formed (21, 22).

Perimenopause is an important transitional period in women’s lives. It not only represents the transition of women from middle age to old age. In fact, it is closely related to the quality of life and physical and mental health of middle-aged and elderly women (23). Perimenopause is a dynamic process. During this process, ovarian function declines continuously. Estrogen secretion decreases gradually. The feedback regulatory mechanism of the hypothalamic–pituitary-ovarian axis has an imbalance. This difference will cause a compensatory increase in the levels of follicle-stimulating hormone and luteinizing hormone. Among them, the increase in follicle-stimulating hormone will be greater and more obvious than that of luteinizing hormone. At the same time, the conversion of testosterone and androstenedione increases. Testosterone and androstenedione are androgen precursors. They are converted into estrogen. This conversion causes a significant decrease in endogenous androgen levels (24). Most women have no obvious symptoms. This is due to the regulation and compensation of the body’s autonomic nervous system. Some women may experience symptoms such as hot flashes, sweating, insomnia, depression or irritability, which is called perimenopausal syndrome (25, 26). In recent years, many women tend to enter perimenopause early. The reasons are the fast pace of life and high work pressure (27), The accelerated development of DE in perimenopausal women may be related to this situation.

## 3 Potential risk factors for DE in perimenopausal women

### 3.1 Sex hormone decline

#### 3.1.1 The impact of sex hormones on meibomian glands

With age, meibomian glands undergo degenerative changes. These changes include acinar atrophy, keratinization of duct openings, proliferation of surrounding collagen, and reduced innervation (28). These age-related changes combined with sex hormone decline can lead to abnormalities. The abnormalities refer to the quantity and composition of meibum secretion in perimenopausal women. As target tissues of sex hormones, meibomian glands have a large number of sex hormone receptors. They also have the basic enzymes needed to produce and break down the sex hormone (29). Perimenopausal women have a significantly increased risk of MGD, which may be closely related to the decline in sex hormone level (30).

Androgen is an extremely important substance that can control the growth, development, differentiation and lipid production of sebaceous glands throughout the body. In fact, the meibomian glands are also controlled and regulated by androgen mediation (31)^.^ Currently, there is a view that androgen deficiency is a factor leading to MGD (32). Clinical studies show that postmenopausal women with severe evaporative DE have significantly reduced total serum testosterone (33). The secretion volume of meibomian glands is related to the diameter of the glandular opening and also to the concentration of serum testosterone, both showing a positive correlation (34). Androgens can significantly regulate genes in meibomian glands. These genes are related to lipid metabolism and keratin synthesis. Through this regulation, androgens participate in the synthesis and secretion of meibum and the inhibition of hyperkeratosis of keratin in the meibomian duct epithelium (35). Androgen deficiency leads to an increased proportion of free fatty acids (FFA) in meibum. These FFAs undergo saponification to form irritating foam-like secretions. These secretions accumulate on the eyelid margin, induces inflammation of the ocular surface and destroys the stability of the tear film (36, 37). Hyperkeratinization of duct cells is regarded as one of the main initiating factors causing obstructive meibomian gland dysfunction. Androgens can regulate the expression of multiple genes. These genes are related to the keratinization process of meibomian glands (38). In addition, androgens reduce ocular surface inflammation by inhibiting the expression of inflammation-related genes such as MMP-9 (39). The decrease in androgen levels during perimenopause may lead to dysfunction of meibomian glands.

The impact of estrogen on meibomian gland function is not fully clear. Estrogen may contribute to the development of MGD by inhibiting meibum synthesis and secretion, as well as impairing the proliferation of meibomian gland epithelial cells (40, 41). The proposed mechanisms include competitively antagonizing the androgen receptor (42), downregulating genes involved in lipid synthesis and secretion within the meibomian glands (43), interfering with the cAMP signaling pathway (43) and other means. Patients with polycystic ovary syndrome (PCOS) receiving estrogen therapy experience almost irreversible loss of meibomian glands. This finding further supports the potential negative effects of estrogen (44). However, some studies indicate that estrogen therapy has an improving effect on MGD in perimenopausal women (30). Estrogen can, to a certain extent, regulate the expression of genes related to lipid and steroid synthesis, as well as immune responses to a certain extent (43). In addition, estrogen shows protective effects such as inhibiting ocular surface inflammatory responses (45), reducing epithelial cell apoptosis (46), and promoting the synthesis of lipoproteins in tears (47). This dual effect may be related to different biological effects. These effects are produced after the activation of different estrogen receptor subtypes (ERα and ERβ) (48).

In conclusion, MGD in perimenopausal women may be mainly attributed to a sharp decline in the effect of androgens on promoting meibomian fat secretion.

#### 3.1.2 The impact of sex hormones on lacrimal glands

The lacrimal gland is actually a crucial part of the LFU. It is responsible for the production and secretion of tears. This process involves precise regulation by multiple systems. The content of acini in the lacrimal gland tissue of elderly women is relatively low, while the content of connective tissue and fat is relatively high. Some age-related degenerative changes (such as lymphocyte infiltration, acinar atrophy, and fibrosis around acini and ducts) progress more significantly compared to elderly men of the same age (49).

Androgens play a particularly crucial regulatory role in the normal function of the lacrimal gland and have an undeniable impact on maintaining its normal operation. Androgen receptors are widely distributed in the acini and epithelial cells of the lacrimal gland ducts (50, 51). In the lacrimal glands of mice, androgens can control the expression of approximately 2,000 genes, and affect the morphological development, cell differentiation and secretion function of the lacrimal gland (52–54). Androgen deficiency can directly cause dysfunction of the lacrimal glands. This functional disorder can lead to the development of water deficiency DE (55, 56). In the Sjogren syndrome mouse model, androgens can effectively inhibit the occurrence of inflammation in the lacrimal glands. They also improve lacrimal gland function. They achieve this by regulating immune-related gene pathways (57). Considering the potential mechanisms, subcutaneous administration of Dihydrotestosterone (DHT) can increase the expression level of TGF-*β*, and reduce the number of inflammatory factors, among which IL-1 β and TNF-*α* are included. Thus, it alleviates inflammatory stimulation (58).

Studies on the impact of estrogen on lacrimal glands have not reached a consensus. Some clinical studies and animal studies show that hormone replacement therapy (HRT) can promote tear secretion in postmenopausal women (59, 60), increase tear peroxidase activity (61). It also shows certain anti-inflammatory effects and anti-apoptotic effects to protect lacrimal gland cells (58, 62, 63). On the contrary, some studies indicate that estrogen has negative effects on lacrimal glands. The use of HRT on menopausal women, may increase the possibility of them developing DE (64). For example, Sjögren’s syndrome mice receive 17 *β*-estradiol treatment. After the treatment, their tear volume decreases significantly (65). These conflicting findings may be associated with the presence of enzymes necessary for the synthesis and metabolism of sex hormones within the lacrimal gland (66). This actually means that the lacrimal glands have the ability to synthesize and secrete sex hormones in local areas of the body.

#### 3.1.3 The impact of sex hormones on the cornea and conjunctiva

Androgens play a crucial protective role in the conjunctiva and cornea. To achieve such a protective effect, androgens rely on promoting cell proliferation and secretion, inhibiting inflammatory responses, and facilitating tissue repair (11). Researches show that, on average, the number of goblet cells in the conjunctiva of men is slightly higher than that of women. The number of goblet cells in women fluctuates with the menstrual cycle. This suggests that goblet cells are regulated by sex hormones (67). Within the molecular scale range, DHT has the ability to regulate the expression of many genes in immortalized human conjunctival epithelial cells. It promotes development, regeneration and migration processes. At the same time, it inhibits immune responses and mitosis (68). Further studies confirm that DHT can inhibit LPS-induced LTB4 secretion and the upregulation of pro-inflammatory factor gene expression caused by hyperosmotic stress (67, 69, 70). Animal experiments also show that the ultrastructure of corneal epithelium in castrated mice is damaged. Exogenous DHT supplementation can effectively improve their corneal morphology and tear film function (56).

During a woman’s menstrual cycle and pregnancy, the level of estrogen can change, which can cause fluctuations in the sensitivity and thickness of the cornea. This indicates that estrogen may play a relatively crucial role in maintaining the stability of the cornea (71, 72). *In vitro* experiments proved that estrogen treatment can down-regulate the mRNA expression of inflammatory factors such as IL-6, IL-8 and TNF-*α* in human corneal epithelial cells (45, 73). In the rat model with ovaries removed, estrogen deficiency can lead to a reduction in tear secretion and also cause abnormal elevation of the corneal epithelium, while estrogen supplementation can reverse the above changes, which may be related to the up-regulation of MMP-2 expression (74). 17β-estradiol can also promote the proliferation and migration of corneal epithelium by enhancing the expression of corneal epidermal growth factor (EGF), thereby accelerating corneal wound healing (75). Clinical studies show that 17β-estradiol eye drop treatment and HRT can improve the symptoms of keratoconjunctivitis sicca in postmenopausal women (76–78). They also increase the proportion of mature corneal cells and the density of goblet cells.

**Figure 1:**
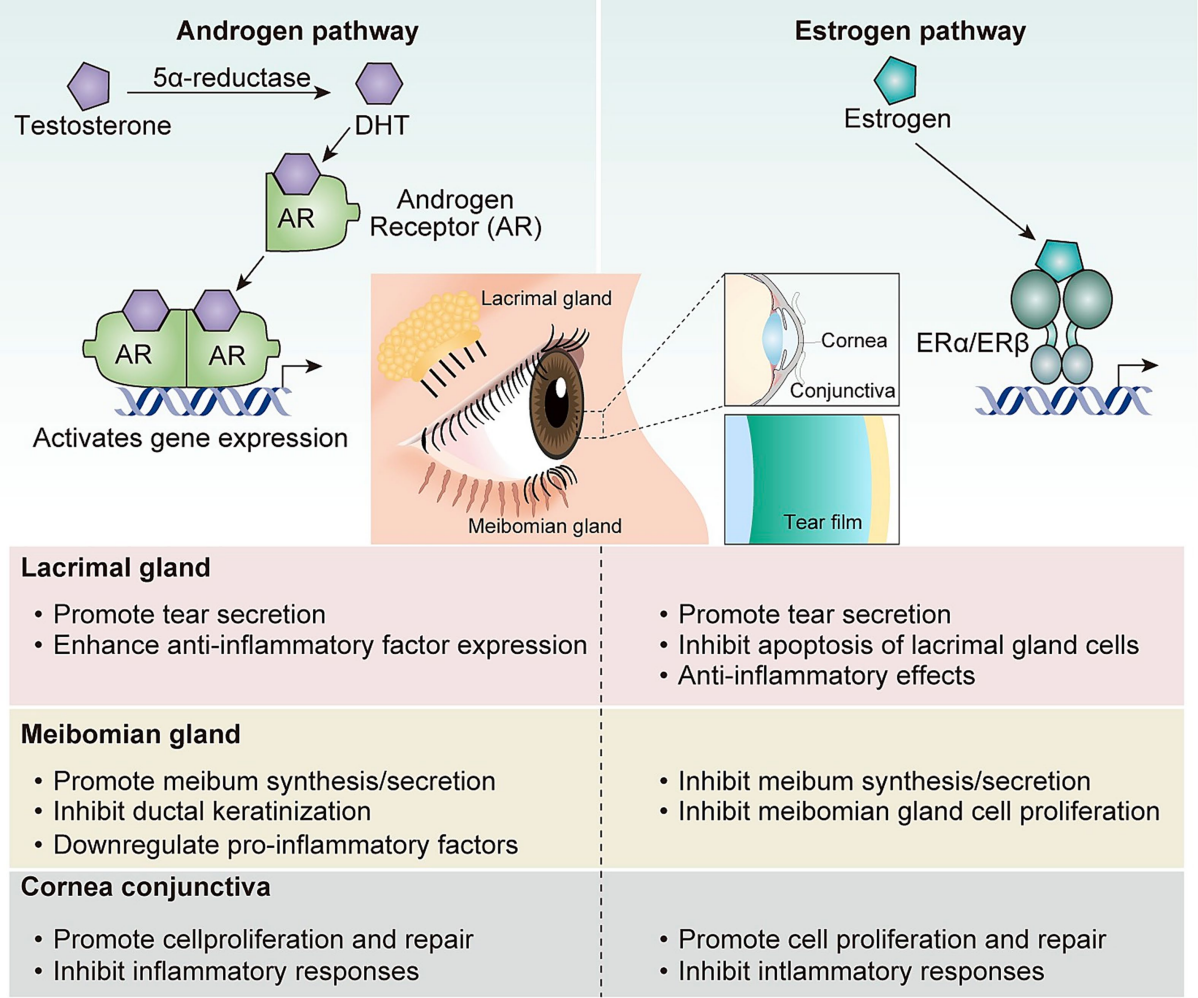
Regulatory role of sex hormones on the lacrimal functional unit.

In summary, both androgens and estrogens play important regulatory roles in various components of the LFU (Table 1). Their effects are both complex and often antagonistic to each other. This imbalance between hormones, together with the hormonal environment dominated by androgen decline, constitute the physiological and pathological basis for the increased susceptibility to DE in perimenopausal women (Figure 1). It is important to note that inconsistencies in studies regarding the ocular effects of estrogen may be partly due to several factors. These include differences in estrogen sources (endogenous vs. exogenous), formulation types (such as oral or percutaneous), and baseline subject characteristics (including the presence of concurrent autoimmune diseases). Therefore, more targeted clinical studies are neeDE in the future to better understand the actual impact of estrogen on the ocular surface.

**Table 1:** Regulatory effects of androgens and estrogens on the lacrimal functional unit.

Hormone	Target site	Main mechanisms and effects	Overall effect
Androgen	Meibomian gland	Promotes synthesis and secretion of meibum (35); Inhibits keratinization of duct epithelium (38); Downregulates expression of pro-inflammatory factors (39)	To protect meibomian gland function and reduce evaporative dry eye
	Lacrimal gland	Promotes tear secretion (50, 54); Expression of anti-inflammatory factors, inhibits inflammation (58)	Improves tear secretion; exerts anti-inflammatory effect to protect lacrimal gland
	Cornea	Promotes proliferation and repair of corneal epithelial cells (11); Inhibits inflammatory response (67, 69, 70)	Maintains integrity of corneal structure and function
	Conjunctiva	Promotes goblet cell proliferation and mucin secretion (68); Inhibits the expression of inflammatory cytokines (69)	Enhances stability of the mucin layer
Estrogen	Meibomian gland	Inhibits lipid synthesis and secretion by antagonizing androgen receptors or interfering with cAMP pathway (40–43)	Inhibits meibum secretion in most cases; may exacerbate MGD
	Lacrimal gland	Protective effects: Promotes tear secretion, anti-apoptosis, anti-inflammation (59, 62, 63). Adverse effect: Inhibits tear secretion (65)	Effects are complex; may be bidirectional or dependent on receptor subtypes (ERα/ERβ)
	Cornea	Inhibits inflammatory response (45, 73). Promotes migration of epithelial cells and wound healing (74, 75)	Exerts anti-inflammatory and repair effects in most cases
	Conjunctiva	Increases goblet cell density (77, 78). Inflammatory response (76)	May have protective effects, especially in local application

### 3.2 Other secondary and synergistic risk factors

#### 3.2.1 Immune dysfunction and enhanced inflammatory response

There is a rather complex connection between the occurrence and development of DE and inflammation. Due to factors such as ovarian function decline, fluctuation of hormone levels and psychological stress, perimenopausal women are prone to endocrine disorders and immune system disorders (79). Chronic stress, in particular, has been shown to induce profound immune dysregulation through neuroendocrine-immune interactions, potentially amplifying inflammatory responses at the ocular surface (80). The manifestations include enhanced infectious inflammatory response and increased probability of autoimmune diseases. Generally, within the same age group, women tend to have a more robust immune response compared to men (81, 82). After menopause, the number of pro-inflammatory cytokines in women’s blood will increase significantly. This trend is consistent with the decline in sex hormones and HRT can partially reverse this process (83, 84). With the emergence of this change, the number of CD4^+^ T cells and B lymphocytes also declined, and the ratio of CD4^+^ to CD8^+^ T cells reversed (85, 86). The mechanism may be related to estrogen receptor signal transduction, nitric oxide activity, and the regulation of antioxidant capacity (83).

Immune homeostasis imbalance and chronic subclinical inflammation are the initiating links of chronic inflammatory diseases such as DE. Without effective control, pro-inflammatory factors will prompt immune cells to move and penetrate to the surface of the eye, leading to ocular barrier destruction, nerve sensitization and gland secretion dysfunction (87, 88). An overly exaggerated inflammatory response may also lead to a decline in corneal nerve innervation. After the decline in corneal nerve innervation, corneal sensitivity will decrease, and the reflex function will also weaken. It results in excessive tear evaporation and decreased lacrimal gland stability (89). In addition, the decline in antioxidant capacity in perimenopausal women can participate in the occurrence and development of DE by promoting lipid peroxidation and inflammatory cell infiltration (90), inducing oxidative damage of lacrimal gland (91), and inhibiting corneal epithelial cell regeneration (92).

Among many autoimmune diseases, such as Sjogren’s disease and systemic lupus erythematosus, the probability of women getting these diseases is significantly higher than that of men (93). Similarly, women have a higher incidence of immune-related dry eye (IR-DE). They also show more severe clinical manifestations and ocular surface inflammation (94). This difference may originate from the immunomodulatory effect of sex hormones. The dramatic fluctuations in sex hormones during perimenopause further amplify immune dysfunction. This makes this group a high-risk population for IR-DE. Mechanistically, the abnormally activated immune system can not only directly attack lacrimal gland tissue (95). It can also indirectly damage the LFU and destroy the ocular surface barrier by enhancing the ocular inflammatory response (96). Multiple mechanisms overlap with each other. Finally, it leads to a serious imbalance of ocular surface homeostasis.

In summary, perimenopausal sex hormone decline weakens the inhibition of NF-κB and other pro-inflammatory pathways (97), leading to chronic systemic and ocular surface inflammation. Inflammatory factors destroy the function of lacrimal gland, meibomian gland and ocular surface barrier, leading to tear hyperosmolarity and tear film instability (98), which further activates immune cells, exacerbates inflammatory response, and finally forms a vicious cycle of “sex hormone-inflammation-tissue damage”, continuously driving the progress of perimenopausal DE.

#### 3.2.2 Perimenopausal depression

The ratio of major depression is 2:1 when comparing women to men, and it is thought that women are more vulnerable to stressors and discrimination throughout development into adulthood (99). Perimenopausal women often face multiple pressures. These pressures come from career, family and physiological changes. They are prone to negative emotions such as depression and anxiety. According to statistical data, the incidence of depression among middle-aged women worldwide is as high as 43.34% (100, 101). Recent studies have shown that there is actually a two-way relationship between DE and depression. They are causes and effects of each other (102). Some studies have found that the prevalence of depression and anxiety in DE patients can reach 40 and 39%, respectively, and the scores of anxiety and depression in DE patients are significantly higher than those in healthy people (102). On the contrary, compared with healthy controls, depressed patients also show shorter tear break-up time (TBUT) and more severe dry eye symptoms, which were more significant in patients with major depression patients (103).

The mechanism underlying the susceptibility of depressed patients to DE is not yet clear. Possible reasons include the following points. (1) The use of antidepressant drugs. Common antidepressants (such as selective serotonin reuptake inhibitors) have anticholinergic effects. They can inhibit lacrimal gland secretion and promote ocular surface inflammation. Thus, they promote the occurrence of DE (104, 105). (2) The lack of serotonin. Serotonin abnormalities related to depression can also affect tear secretion. There is a complex connection between the concentration of serotonin in tears and the manifestations and indications of DE (106, 107). (3) Elevation of proinflammatory cytokines. In the tears of those suffering from depression, the levels of pro-inflammatory cytokines (such as IL-6, IL-17, and TNF-*α*) have risen. This situation is likely to make the inflammation on the ocular surface more severe and also cause tissue damage (108). (4) The disorder of neural regulation function. Depression is often accompanied by central sensitization and hypothalamic–pituitary–adrenal axis (HPA) disorder. This makes patients have lower pain threshold and higher corneal sensitivity. Then it leads to abnormalities in the quality or quantity of tears (109, 110).

#### 3.2.3 Sleep disorder

Sleep Disorder refers to a clinical syndrome. It is characterized by persistent difficulties in the initiation, maintenance, duration or quality of sleep. It leads to significant impairment of daytime function. About 33 to 51% of perimenopausal women have sleep disorders (111). One study has shown that among people with relatively short sleep duration and those with severe sleep interruptions, the incidence of DE is 1.2 times and 1.29 times that of those with optimal sleep conditions (112). In animal models, sleep deprivation causes typical DE characteristics in mice. These characteristics include reduced tear secretion, increased corneal sensitivity, corneal epithelial cell damage and increased cell apoptosis (113). Sleep deprivation can induce DE by causing hyperosmolarity of tears and reducing tear secretion. This may be related to increased cortisol, adrenaline and noradrenaline. These increases are caused by insufficient sleep and poor sleep quality. It may also be related to reduced parasympathetic tone (114). Insufficient sleep can activate the hypothalamic–pituitary–adrenal axis. Moreover, it can disrupt the circadian rhythm of the renin-angiotensin-aldosterone system. Thus, it promotes the excretion of sodium ions and water by the kidneys (115). These systemic hormonal changes plus relative dehydration will further affect the production of tears.

#### 3.2.4 Use of hormone replacement therapy

Hormone replacement therapy (HRT) is an effective means to relieve perimenopausal syndrome and prevent osteoporosis (116). Over the past few years, a large number of research projects have demonstrated that the use of HRT may increase the likelihood of people developing DE. In a relatively large cohort study project, postmenopausal women who receive estrogen or a combination of estrogen and progesterone have an increased risk of being diagnosed with DE or developing severe dry eye symptoms by approximately 70 and 30%, respectively (64). A separate cross-sectional survey was conducted, which revealed a very significant difference in the severity of DE between postmenopausal women receiving HRT and controls, and long-term HRT use appeared to increase the risk of DE (117). The exact mechanism by which HRT increases the risk of DE has not been fully clarified. It may be related to exogenous estrogen inhibiting meibum secretion (43), the antagonism of androgen protective effect (118), and the indirectly aggravating ocular surface inflammation and disrupting tear film homeostasis (65). At present, the conclusions of studies on the correlation between HRT and DE are inconsistent. Some studies report neutral or even protective results (Table 2), which may be related to the HRT regimen (single or combined), administration route (oral vs transdermal), differences in dosage and course of treatment, as well as the genetic background, underlying diseases and baseline ocular surface status of the study population are closely related. In summary, at the level of DE risk assessment, the available evidence does not support the development of uniform guidelines for the clinical application of HRT in perimenopausal women. In the future, more targeted studies are needed to establish clinical practice guidelines that can identify high-risk individuals in this group, improve overall menopausal health, and prevent and control DE.

**Table 2:** A summary of relevant research on the impact of hormone replacement therapy on dry eye in postmenopausal and perimenopausal women.

Study (year)	Study participants (*n*)	Study type	Main tear/Ocular surface outcomes	Main findings	Key conclusions/Notes
Li et al. (2025) (121)	Perimenopausal women (*n* = 100)	RCT	OSDI; TBUT; Schirmer’s test; CFS	Estrogen replacement therapy combined with autologous serum showed significantly better improvements in all indicators than autologous serum alone.	Estrogen replacement therapy combined with autologous serum for the treatment of severe DE in perimenopausal women can achieve very good effects.
Golebiowski et al. (2017) (122)	Postmenopausal women with dry eye symptoms (*n* = 38)	RCT	OSDI; Tear osmolarity; TBUT; Schirmer’s test	After 8 weeks of treatment, the symptoms of dry eye in the estrogen group were significantly more severe than those in the control group.	Short-term single hormone therapy is ineffective and even harmful.
Feng et al. (2016) (60)	Postmenopausal women (*n* = 88)	Prospective Cohort Study	Schirmer’s test	The Schirmer value in the HRT group was significantly higher than that in the control group for women aged 40–55; it was significantly lower for women aged 56–70.	The impact of HRT on dry eye is significantly age-dependent.
AlAwlaqi et al. (2016) (117)	Postmenopausal women (*n* = 360)	Cross-Sectional Study	OSDI; Tear osmolarity	In the HRT group, a larger proportion of individuals have developed DE, and the symptoms they exhibit are more obvious.	HRT is a factor that can cause the severity of DE. The risk of treatment with estrogen alone is greater than that of combined treatment.
Scuderi et al. (2012) (123)	Postmenopausal women with DE (*n* = 66)	Cross-Sectional Study	Tear osmolarity; Schirmer’s test; TBUT	Oral phytoestrogens significantly improved tear osmolarity, secretion volume and stability.	Phytoestrogens are a potential treatment option for DE in postmenopausal women.
Coksuer et al. (2011) (124)	Postmenopausal women (*n* = 34)	RCT	OSDI; Schirmer’s test; TBUT	After 6 months of treatment with drospirenone + progesterone, OSDI score decreased significantly, Schirmer value increased significantly, and TBUT prolonged significantly.	Specific estrogen-progestin combination therapies containing drospirenone have significant benefits in improving dry eye signs and symptoms in postmenopausal women
Piwkumsribonruang et al. (2010) (125)	Postmenopausal women with DE (*n* = 42)	RCT	Symptom score; Schirmer’s test; TBUT	The HRT group had significantly improved symptom scores and prolonged TBUT, but no significant difference in Schirmer value.	HRT improves symptoms and tear film stability, but does not increase basic tear secretion.
Erdem et al. (2007) (126)	Postmenopausal women (*n* = 80)	Cross-Sectional Study	Schirmer’s test; TBUT; Corneal Fluorescein Staining	Long-term HRT users (>3 years) showed worse TBUT and corneal fluorescein staining scores.	The adverse effects of HRT on DE may be related to the duration of its use.
Khurana et al. (2006) (127)	Postmenopausal women with DE (*n* = 11)	Prospective Study	IOP	HRT led to a significant increase in IOP (from 14.5 ± 2.5 mmHg to 17.3 ± 2.5 mmHg).	Estrogen-androgen combined therapy may lead to a significant increase in intraocular pressure in postmenopausal women.
Uncu et al. (2006) (128)	Postmenopausal women (*n* = 30)	Prospective Cohort Study	Schirmer’s test; IOP	The Schirmer value in the HRT group decreased significantly, and the intraocular pressure in the pure estrogen treatment group decreased significantly.	HRT can lead to a reduction in tear secretion, and the duration of HRT use is positively correlated with the risk of dry eye
Kuscu et al. (2003) (129)	Postmenopausal women (*n* = 60)	Prospective Cohort Study	Schirmer’s test; TBUT; Rose Bengal Staining	The Schirmer value in the HRT group decreased significantly, with no significant differences in TBUT and Rose Bengal Staining scores.	Short-term combined HRT treatment has a significant inhibitory effect on the basic tear secretion function of postmenopausal women.
Okońet al (2001) (130)	Perimenopausal and postmenopausal women (*n* = 60)	Prospective Cohort Study	Schirmer’s test; TBUT	The Schirmer value in the HRT group increased significantly, and TBUT prolonged significantly.	HRT can effectively increase tear secretion and improve tear film stability, with more significant efficacy in postmenopausal women.
Schaumberg et al. (2001) (64)	Postmenopausal women (*n* = 25,665)	Cross-Sectional Survey	Prevalence of DE	Women using estrogen alone or estrogen + progesterone had a significantly higher risk of DE. Women using HRT for ≥10 years had the highest risk.	HRT significantly increases the risk of DE, especially in those using pure estrogen or long-term HRT.

HRT, Hormone Replacement Therapy; RCT, Randomized Controlled Trial; OSDI, Ocular Surface Disease Index; TBUT, tear break-up time; CFS, corneal fluorescein staining; IOP, intraocular pressure.

## 4 Conclusion

DE is a common chronic ocular surface disease with a high prevalence worldwide. Its pathogenic mechanism is complex. Its disease course is chronic. These factors make most patients only able to relieve and control symptoms through medical means. It is difficult to cure DE. DE in perimenopausal women is characterized by high incidence, complex pathological mechanism, and multiple influencing factors. This review summarizes the epidemiological trends and pathological mechanism of DE. On this basis, it focuses on discussing multiple susceptibility factors of DE in perimenopausal women.

**Figure 2:**
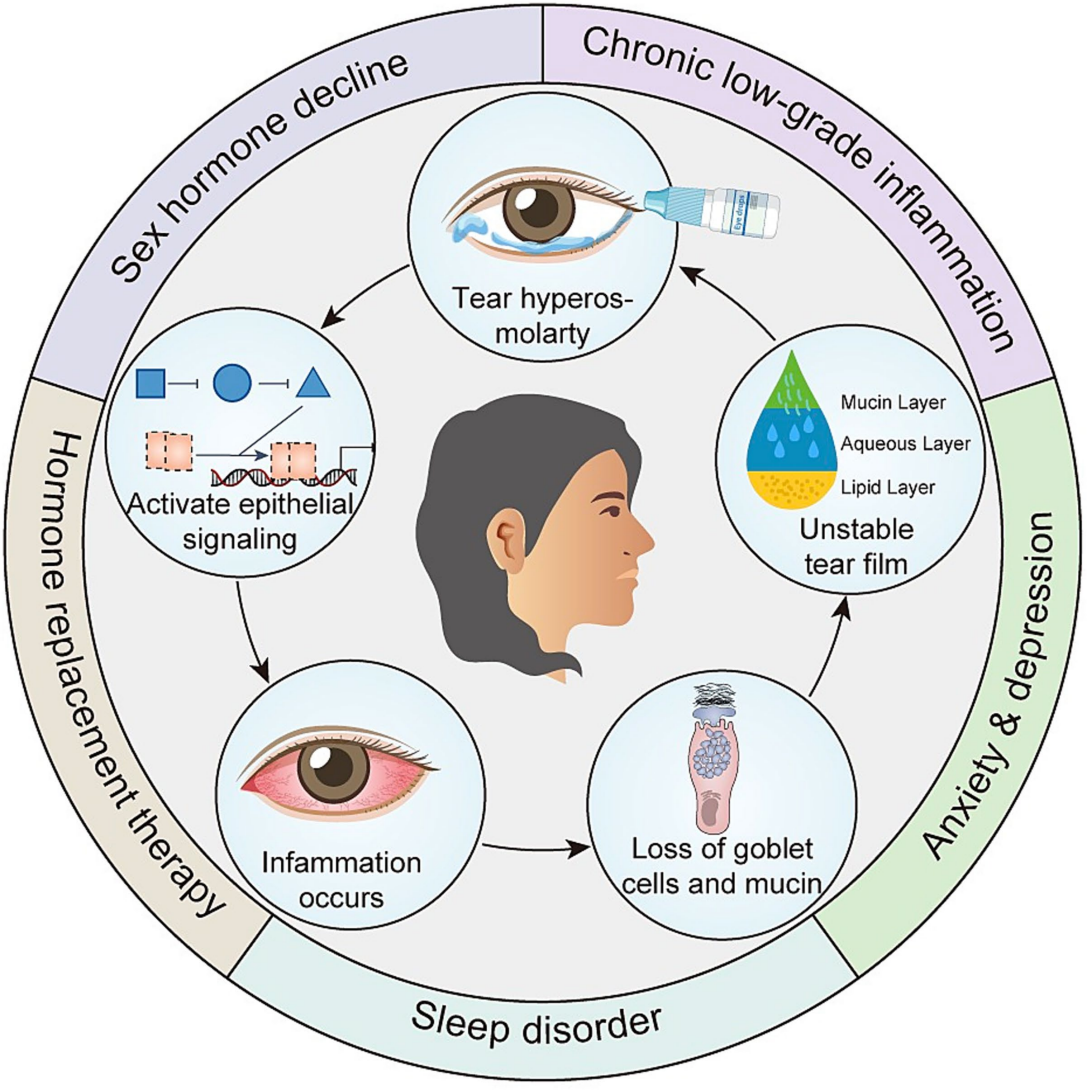
Multifactorial synergistic pathogenesis of dry eye in perimenopause.

Sex hormone axis disorder constitutes the biological basis for the occurrence of DE in perimenopause. Sex hormones can directly or indirectly regulate various components of the LFU. They have a practical impact on the quality, quantity and kinetics of the tear film. Sex hormone disorders can trigger and sustain a chronic low-grade inflammatory state. This occurs because pro-inflammatory cytokines are upregulated while the expression of anti-inflammatory cytokines is inhibited. This persistent inflammatory response is actually a key factor that disrupts the balance of the eye surface. Eventually, a vicious cycle of “sex hormone imbalance-inflammation-tissue damage” will be formed. The common perimenopausal symptoms—including anxiety, depression, and sleep disturbances—arise from declining sex hormone levels and can exacerbate both systemic and ocular surface inflammation. At the same time, the negative impact of these symptoms on the treatment compliance and quality of life of patients will further increase the discomfort symptoms of DE and the difficulty of management. In addition, HRT is used to relieve perimenopausal symptoms. It itself may also increase the risk of adverse reactions such as ocular dryness (Figure 2). In short, the occurrence of DE during perimenopause is the result of the combined effect of endogenous physiological changes (centered on the decline of sex hormones) and exogenous environmental and behavioral factors. These risk factors do not act independently but rather interweave and reinforce each other, generating a synergistic and amplifying effect. This suggests that the prevention and treatment of DE in perimenopausal women should adopt a highly individualized and comprehensive management strategy.

It must be admitted that this review also has certain limitations. At present, there is no consensus on the impact of sex hormones on DE. The differences in research results may be due to the variations in design among different studies. This could be because sex hormones have relatively complex biological effects. Additionally, DE itself is heterogeneous, which could also be the cause of these inconsistent conclusions. Moreover, the bidirectional regulatory effect of sex hormones, the specificity of receptor subtypes (ERα/ERβ), the synergistic/antagonistic effects with other hormones, and the differences in concentration and activity between local and circulating hormones all increase the difficulty of explaining its mechanism. Secondly, DE animal models (such as mice, rabbits, dogs, etc.) that are widely used at present are species different from humans in terms of ocular tissue structure and tear composition. For example, compared with humans, the acinus of mice and rats is closely packed, the acinar lumen is small and highly invaginated, and the major component of rabbit meibomian fat is dominated by saturated fatty acids. These differences limit the translation of relevant research conclusions to clinical practice. Finally, some definitions related to perimenopause, DE, and systemic diseases varied among the included studies, which may cause potential and uncontrolled bias.

In conclusion, a deep understanding of the risk factors and related molecular mechanisms of DE in perimenopausal women will help to build a more targeted and cost-effective prevention and control system. In the future, artificial intelligence technology will play an increasingly important role in the diagnosis and treatment of perimenopausal DE (119, 120), which is expected to further improve the precision diagnosis and treatment of perimenopausal DE, thereby improving the ocular surface health of this population and reducing the overall disease burden of DE.
